# Serum and Extracellular Vesicle MicroRNAs miR-423, miR-199, and miR-93* As Biomarkers for Acute Graft-versus-Host Disease

**DOI:** 10.3389/fimmu.2017.01446

**Published:** 2017-11-10

**Authors:** Rachel E. Crossland, Jean Norden, Mateja Kralj Juric, Kim F. Pearce, Clare Lendrem, Louis A. Bibby, Matthew Collin, Hildegard T. Greinix, Anne M. Dickinson

**Affiliations:** ^1^Medical School, Institute for Cellular Medicine, Newcastle University, Newcastle upon Tyne, United Kingdom; ^2^Department of Internal Medicine I, Medical University of Vienna, Vienna, Austria; ^3^Division of Hematology, Medical University of Graz, Graz, Austria

**Keywords:** graft-versus-host disease, biomarkers, prognosis, microRNA, extracellular vesicles

## Abstract

Acute graft-versus-host disease (aGvHD) is a major cause of adverse outcome in hematopoietic stem cell transplantation (HSCT), with a high incidence (20–50%). A novel, non-invasive diagnostic test to predict for prevalence and severity would enable improved prophylaxis and reduce morbidity. Circulatory microRNAs (miRNAs) miR-423, miR-199, miR-93*, and miR-377 have previously been associated with aGvHD in post-HSCT patient plasma, but validation is lacking and their expression within extracellular vesicles (EVs) has not been explored. This study replicated elevated serum expression of miR-423 (*p* < 0.001), miR-199 (*p* = 0.04), miR-93* (*p* < 0.001), and miR-377 (*p* = 0.03) in aGvHD, using a prognostic cohort of day 14 (D14) post-HSCT patient samples (*n* = 81). Expression also associated with disease severity. Further analysis at aGvHD diagnosis in an independent cohort (*n* = 65) confirmed high miR-423 (*p* = 0.02), miR-199 (*p* = 0.007), and miR-93* (*p* = 0.004) expression at disease onset. Investigation of expression patterns during early HSCT sequential timepoints (pre-HSCT to D28) identified elevated miRNAs at D7 post-HSCT in all transplant patients. In a novel investigation of miRNA expression in serum EVs (*n* = 15), miR-423 (*p* = 0.09), miR-199 (*p* = 0.008), and miR-93* (*p* = 0.001) levels were lower at D14 in patients who later developed aGvHD, and this was replicated for miR-423 (*p* = 0.02) and miR-199 (*p* = 0.04) (*n* = 47). Comparing serum to circulating EVs, at D14 patients remaining aGvHD-free had higher expression of miR-423 (*p* = 0.03), miR-199 (*p* = 0.009), and miR-93* (*p* = 0.002) in the EV fraction. Results verify the capacity for circulating miR-423, miR-199, and miR-93* as diagnostic and prognostic aGvHD biomarkers. The novel finding of their differential expression in EVs suggests a potential role in aGvHD etiology.

## Key Points

- Expression of miR-423, miR-199, and miR-93* in post-HSCT serum and extracellular vesicles is associated with aGvHD incidence and severity.
- Differential expression in extracellular vesicles suggests these microRNAs may be implicated in the pathology of aGvHD.

## Introduction

Graft-versus-host disease (GvHD) is a major cause of morbidity and mortality in hematopoietic stem cell transplantation (HSCT). Despite recent advances, the incidence remains high (20–50%) and GvHD therapy exacerbates infectious complications. Acute GvHD (aGvHD) classically develops in the first 3 months post-HSCT and can almost double the cost of a transplant. Thus, a novel, non-invasive, diagnostic test that predicts the incidence and severity of aGvHD would enable more timely prophylactic therapy, reducing morbidity, and health care costs.

MicroRNAs (miRNAs) have been demonstrated as informative biomarkers for various diseases including inflammatory conditions and cancer. miRNAs are small, non-coding single-stranded RNAs, and regulate approximately 50% of all genes, by binding to the 3′UTR and repressing translation (1). More recently, miRNAs have been identified in bodily fluids where they are protected from RNase-mediated degradation by encapsulation into extracellular vesicles (EVs) or through binding to protective proteins [reviewed in Cortez et al. (2)]. These miRNAs demonstrate a novel capacity to regulate the cellular differentiation of blood cells and immune function (3). Furthermore, distinct miRNA expression patterns are associated with specific pathophysiological conditions (4).

With regard to GvHD, miRNAs represent ideal candidates for biomarker identification as they can be assessed using accurate and sensitive technology [e.g., quantitative real-time PCR (qRT-PCR)], quantified in bodily fluids that require minimally invasive sample collection (e.g., serum/urine), and further investigated for biological function (e.g., target protein identification) that may expand upon our understanding of GvHD biology. Although the field of GvHD-related miRNA and EV research is in its infancy, recent studies have demonstrated an emerging role for miRNAs as GvHD biomarkers. Indeed, miR-155, an immune cell-related miRNA, is up-regulated in T-cells from mice developing aGvHD following HSCT, and miR-155 inhibitors resulted in decreased aGvHD severity and prolonged survival (5). Furthermore, recent murine models have shown that T regulatory cells (Tregs), critical cells in the prevention of auto-immunity, can modulate effector T-cell responses *via* the release of EV encapsulated miRNAs (6).

Xiao et al. previously reported that a four miRNA plasma signature (miR-423, miR-199, miR-93*, and miR-377) could predict the probability of aGvHD and was also elevated at a median of 16 days prior to disease onset (7). In addition, the miRNA signature was associated with aGvHD severity and survival (7). However, the data have not been replicated in an independent allogeneic HSCT (allo-HSCT) cohort. Furthermore, serum samples have been shown to demonstrate higher miRNA expression than plasma (8), while expression correlates well between the two fractions (9), indicating serum may be an alternative non-invasive fluid for biomarker assessment.

This study sought to replicate the prognostic and diagnostic potential of miR-423, miR-199, miR-93*, and miR-377, by assessing expression in serum samples from HSCT patients taken prior to disease onset [day 14 (D14), prognostic cohort], or at disease presentation (date of diagnosis, diagnostic cohort). To further explore the expression patterns of these miRNAs during early HSCT, expression was assessed in serum samples taken at sequential time points from pre-transplant to day 28 post-HSCT. Finally, a novel investigation of miRNA expression in the EV fraction of serum was performed at sequential, prognostic, and diagnostic time points, in order to elucidate their potential role in circulating microvesicles during aGvHD biology.

## Materials and Methods

### Clinical Samples and Ethics

The initial prognostic cohort comprised 81 allo-HSCT patient serum samples taken at D14 post-HSCT, prior to the onset of aGvHD diagnosis. Sequential serum samples (D7 pre-HSCT, D0, D7, D14, and D28) were available for a subset of 34 patients (sequential serum cohort), of which 15 were included in the EV sequential cohort. The EV verification cohort included 47 independent patient serum samples taken at D14 post-HSCT. All patients were transplanted at the Freeman Hospital, Newcastle upon Tyne, UK. A separate diagnostic cohort of serum samples from 65 patients, taken at the time of aGvHD onset or equivalent time point and transplanted at the Medical University of Vienna, Austria were also assessed. Whole blood samples were collected at D14, time of aGvHD onset or equivalent, or at sequential time points in 7 ml vacutainers containing no anti-coagulant from patients undergoing allo-HSCT (years 2008–2013). Clinical details of the cohorts are shown in Table 1. All blood samples were processed immediately upon receipt, whereby they were left to clot, the supernatant centrifuged at 500*g* for 5 min and stored at −80°C. Serum aliquots were centrifuged at 4,500×*g* for 15 min to remove platelets before use.

**Table 1:** Patient clinical characteristics.

	Prognostic serum cohort (Newcastle)				Diagnostic serum cohort (Vienna)				Sequential EV cohort (Newcastle)				Verification EV cohort (Newcastle)			
Characteristic	All	aGvHD *n* (%)	No aGvHD *n* (%)	*p*-Value	All	aGvHD *n* (%)	No aGvHD *n* (%)	*p*-Value	All	aGvHD *n* (%)	No aGvHD *n* (%)	*p*-Value	All	aGvHD *n* (%)	No aGvHD *n* (%)	*p*-Value
Patients	81	44	37		65	41	24		15	10	5		47	24	23	
Age^a^																
Average	49	46	53	0.09	47	45	49	0.33	48	44	54	0.16	49	49	49	0.93
Range	20–49	20–69	20–69		18–73	18–73	19–71		23–63	23–63	41–61		20–69	20–69	23–68	
Gender^b^																
Male	48 (59)	27 (61)	21 (57)	0.82	36 (55)	26 (63)	10 (42)	0.12	11 (73)	8 (80)	3(60)	0.56	23 (49)	13 (54)	10 (43)	0.56
Female	33 (41)	17 (39)	16 (43)		29 (45)	15 (37)	14 (58)		4 (27)	2 (20)	2 (40)		24 (51)	11 (46)	13 (57)	
Conditioning^b^																
Myeloablative	20 (25)	14 (32)	6 (16)	0.13	25^c^ (44)	19 (53)	6(29)	0.10	3(20)	2(20)	1(20)	1.0	14 (30)	10 (42)	4(17)	0.11
Reduced intensity	61 (75)	30 (68)	31 (84)		32^c^ (56)	17 (47)	15(71)		12 (80)	8 (80)	4(80)		33 (70)	14 (58)	19 (83)	
Relation^b^																
MUD	47 (58)	26 (59)	21 (57)	1.0	43^c^ (75)	31 (78)	12 (60)	0.74	13 (87)	8 (80)	5 (100)	0.52	27 (57)	17 (71)	10 (43)	0.08
SIB	34 (42)	18 (41)	16 (43)		14^c^ (25)	9 (22)	5 (40)		2 (13)	2 (20)	0 (0)		20 (43)	7 (29)	13 (57)	
Survival^b^																
Alive	51 (63)	25 (57)	26 (70)	0.25	57 (88)	35 (85)	22 (92)	0.70	7 (47)	3 (30)	4 (80)	0.12	33 (70)	17 (71)	16 (70)	1.00
Deceased	30 (37)	19 (43)	11 (30)		8 (12)	6 (15)	2 (8)		8 (53)	7 (70)	1 (20)		14 (30)	7 (29)	7 (30)	
Follow up (months)^a^																
Average	11.5	11.8	11.5	0.96	50.9	51.9	49.9	0.99	12.4	14.4	9.1	0.31	11.0	9.7	12.3	0.32
Range	1.2–47.6	2.5–24.7	1.2–47.6		4.1–97.6	4.1–95.0	15.3–97.6		1.4–24.4	2.5–24.4	1.4–15.6		1.2–47.6	2.5–23.5	1.2–47.6	

*Clinical details of the prognostic (*n* = 81) and diagnostic (*n* = 65) serum cohorts, the sequential (*n* = 15) extracellular vesicle (EV), and verification (*n* = 47) EV cohorts to test for differences between acute graft-versus-host disease (aGvHD) versus no aGvHD groups. *p*-Values between groups were calculated using the independent two sample *t*-test^a^ or Fishers Exact test^b^, as appropriate*.

*^c^Some clinical data unavailable*.

*MUD, matched unrelated donor; SIB, sibling donor*.

Patients consented to sample collection and molecular testing and the project was approved by the Newcastle and North Tyneside I Research Ethics Committee and the Ethics Committee of the Medical University of Vienna, Austria. All investigations were conducted in accordance with the Helsinki Declaration. The overall clinical aGvHD grade was established by clinicians in accordance with the NIH consensus and modified Glucksberg criteria (10, 11). All the clinical data were obtained from the EuroTransplantBank database (www.EuroTransplantBank.org).

### Serum Total RNA Isolation

Total RNA was isolated from 250 µl serum using the Norgen Total RNA isolation kit (Norgen Biotek), following the suppliers instructions in Norgen protocol Appendix B.

### EV Precipitation and RNA Isolation

Extracellular vesicles were isolated from 250 µl serum using Life Technologies (LT) total Exosome Isolation Reagent, according to the supplier’s protocols. The EV purity was initially assessed by electron microscopy and nanoparticle tracking analysis, as previously described (12) (Figure S1 in Supplementary Material). All EV pellets were processed immediately for downstream RNA isolation. The EV pellet was initially re-suspended in lysis solution with the addition of 100:1 β-mercaptoethanol. RNA was then isolated using the Norgen Total RNA isolation kit.

### miRNA qRT-PCR

MicroRNA and endogenous control-specific cDNA [miR-423, miR-199-3p, miR-93*, and miR-377, HY3 and U6 (12)] was generated using TaqMan^®^ MicroRNA Assays or TaqMan^®^ Control Assays and the TaqMan^®^ MicroRNA Reverse Transcription kit (LT), according to the supplier’s protocol. Each 15 µl reaction incorporated 4 µl total RNA. qRT-PCR was performed incorporating TaqMan^®^ MicroRNA Assays or TaqMan^®^ Control Assays (LT) and SensiFast Probe Hi-Rox reagent (Bioline). Each 10 µl reaction incorporated 3.25 µl cDNA and thermal cycling was performed in triplicate using the 7900HT Real-Time PCR System (LT), according to manufacturer recommended cycling conditions.

### Statistical Analysis

MicroRNA expression was assessed using SDS2.4 software (LT). Input data were based on converted Δ*Ct* values, according to the comparative *Ct* method (13). Statistical analyses were carried out using SPSS v22.0, Sigmaplot v12.5, or GraphPad Prism v6.0. Graphs were produced using Sigmaplot v12.5 or GraphPad Prism v6.0. Differences between groups were assessed using the Student’s *t*-test (two groups) or one-way ANOVA (less than two groups). Homogeneity of variance was assessed using the Levene statistic. Following a significant ANOVA result, differences between pairs of groups were assessed *via* the Tukey *post hoc* test (SPSS v22). Receiver operating characteristic (ROC) analysis was performed using disease status as the binary state (classification) variable and marker expression on a continuous scale as the test variable (SigmaPlot v12.5). The ROC post-test results used a pre-test prior-probability of 0.5 and cost ratio of 1.0 (14). The optimal cutoff value to dichotomize miRNA expression was computed from sensitivity and specificity using the slope *m* by finding the cutoff that maximizes the function: sensitivity − *m* (1 − specificity) (SigmaPlot v12.5) (15). The accuracy of the test was defined by the area under the curve (AUC) whereby AUC = 0.5 means no diagnostic ability and AUC = 1 means perfect diagnostic ability. Principle components (PCs) were calculated for correlated miRNAs using JMP^®^ Pro v11.2. Correlation between miRNA expression was determined using Pearson’s correlation with Holm–Bonferroni multiple comparisons adjustment applied (16). The first PC1 in all analyses explained the majority of the variance (range 63.5–78.2%) and thus, was used for composite ROC analysis. The weighting used in PC1 for each miRNA was derived from eigenvectors of the correlation matrix. PC1 took the form: “Component 1 = (weighting1 * *Std*miR1) + (weighting2 * *Std*miR2) + …….” where “*Std*miRX = Δ*Ct*_miRx_–mean(Δ*Ct*_miRx_)/SD (Δ*Ct*_miRx_)” (JMP^®^ Pro v11.2). Survival plots were generated using the Kaplan–Meier method and differences in outcome were assessed for significance using the log-rank test (SPSS v22). Cumulative incidence based on the competing risk method, as described by Fine and Gray (17), was used for assessing the association between miRNAs and both relapse and non-relapse mortality (18) using R (R Project) package “cmprsk” (competing risks) (19).

## Results

### Candidate miRNA Expression in Prognostic Serum Samples

The initial prognostic cohort was comprised of serum samples taken at D14 post-HSCT, prior to the onset of symptomatic disease, from *n* = 81 HSCT patients who later developed aGvHD versus no aGvHD [aGvHD *n* = 44 (54%), mean aGvHD onset 42 days post-HSCT]: [grades I = 5 (6%), II = 31 (38%), and III = 8 (10%); no aGvHD *n* = 37 (46%)] (Table 1). Prophylactic therapy comprised of cyclosporine A (CyA) for all patients. No treatment for aGvHD had been given at the time of sampling. There was no significant difference in patient age (*p* = 0.09), gender (*p* = 0.82), conditioning regimen (*p* = 0.13), relationship (*p* = 1.0), survival (*p* = 0.25), or follow up (*p* = 0.96) between patients who developed aGvHD compared with those that did not (Table 1). At the time of follow up, *n* = 16 (20%) patients were relapsed or deceased, *n* = 29 (36%) had NRM, and *n* = 35 (44%) were in remission.

**Figure 1:**
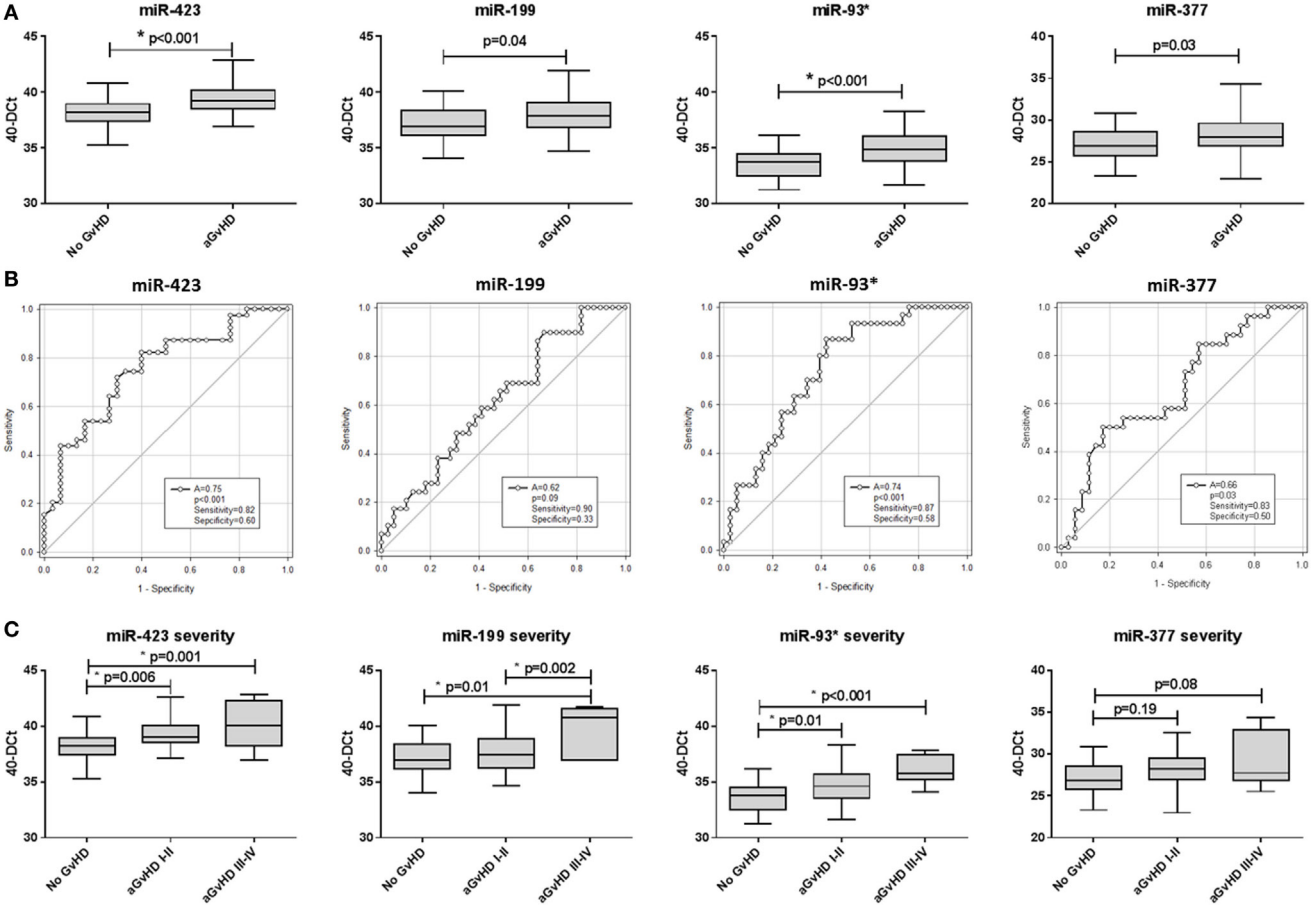
MicroRNA (miRNA) expression in prognostic cohort patient serum samples. Expression of miR-423, miR-199, miR-93*, and miR-377 was assessed by quantitative real-time PCR at day 14 post-hematopoietic stem cell transplantation in the prognostic cohort (*n* = 81) and analyzed according to acute graft-versus-host disease (aGvHD) occurrence and its severity. **(A)** miRNA expression according to aGvHD incidence. Box plot whiskers represent minimum to maximum expression and *p*-values were calculated using the independent two sample *t*-test. **(B)** Receiver operator characteristic curves for incidence of aGvHD to determine prognostic ability of miRNA expression. **(C)** miRNA expression according to aGvHD severity. Box plot whiskers represent minimum to maximum expression and *p*-values were calculated between groups using one-way ANOVA with Tukey *post hoc* multiple comparisons adjustment. *p < 0.05.

Assessing expression of candidate miRNAs, miR-423 (*p* < 0.001), miR-199 (*p* = 0.04), miR-93* (*p* < 0.001), and miR-377 (*p* = 0.03) were expressed at a higher level in patients who developed aGvHD versus no GvHD (Figure 1A). Furthermore, ROC analysis identified miR-423 (*p* < 0.001, AUC = 0.75), miR-199 (*p* = 0.09, AUC = 0.62), miR-93* (*p* < 0.001, AUC = 0.74), and miR-377 (*p* = 0.03, AUC = 0.66) to have prognostic ability with regard to the incidence of aGvHD (Figure 1B). Assessing miRNAs in relation to aGvHD severity, miR-423 (*p* = 0.001), miR-199 (*p* = 0.01), and miR-93* (*p* < 0.001) were expressed at significantly higher levels in severe (III–IV) versus no aGvHD (Figure 1C). miR-423 (*p* = 0.006) and miR-93* (*p* = 0.01) were higher in mild (I–II) versus no aGvHD and miR-199 was significantly higher in severe (III–IV) versus mild (I–II) aGvHD (*p* = 0.002) (Figure 1C). miR-377 expression was not associated with aGvHD grade (Figure 1C). Assessing miRNA expression in relation to clinical factors, there was no significant difference in miR-423, miR-199, miR-93*, or miR-377 comparing male versus female patients or reduced intensity (RIC) versus myeloablative conditioning (*p* > 0.05) (data not shown). miR-423, miR-199, and miR-377 showed no difference in expression comparing sibling versus matched unrelated donors (MUD), while miR-93* expression was higher in patients transplanted from MUD donors (*p* = 0.004) (data not shown).

All four miRNAs demonstrated significant positive correlation in expression (*p* < 0.001–0.031; *R*^2^ = 0.08–0.52) and this remained significant after Holm–Bonferroni multiple testing correction (Figure S2 in Supplementary Material). Due to significant correlation, principle component analysis (PCA) was performed to calculate a composite score. The first PC1, a linear combination of miR-423, miR-199, miR-93*, and miR-377, explained 63.5% of the variability in the data. The resultant score [PC1 = (0.56101 * *Std*miR423) + (0.48296 * *Std*miR199) + (0.53408 * *Std*miR93*) + (0.40839 * *Std*miR377)] was used for ROC analysis and showed good prognostic ability for aGvHD incidence (*p* < 0.001, AUC = 0.74) (Figure S3A in Supplementary Material). There was no significant association with OS (*p* > 0.05), however, patients with a high-PC1 score (≥0.44, the optimal dischotomization threshold identified by ROC analysis) had a significantly higher incidence of NRM (20-month probability of NRM 57 versus 28%, *p* = 0.04) (Figure S3D in Supplementary Material).

### Candidate miRNA Expression in Diagnostic Serum Samples

In order to explore miRNA diagnostic potential, expression was assessed in an independent diagnostic cohort comprising samples taken from patients at the time of aGvHD onset, or equivalent time point for patients who did not develop aGvHD, being treated in a separate Institution (Vienna) and using transplant center-specific treatment protocols. No treatment for aGvHD had been given at the time of sampling. The cohort comprised *n* = 65 HSCT patients [aGvHD *n* = 41 (63%), mean onset 34 days post-HSCT: grades I = 22 (34%), II = 5 (8%), and III = 14 (21%)], no aGvHD *n* = 24 (37%, mean time of sampling 42 days post-HSCT) (Table 1). Prophylactic therapy information was available for 53/65 (82%) patients [CyA + mycophenolate mofetil (MMF) *n* = 24 (45%), CyA + methotrexate (MTX) *n* = 19 (36%), MTX *n* = 7 (13%), MMF *n* = 2 (4%), CyA *n* = 1 (2%)]. There was no significant difference in patient age (*p* = 0.33), gender (*p* = 0.12), conditioning (*p* = 0.10), relationship (*p* = 0.74), or survival (*p* = 0.70), between patients who developed aGvHD compared with those that did not (Table 1).

**Figure 2:**
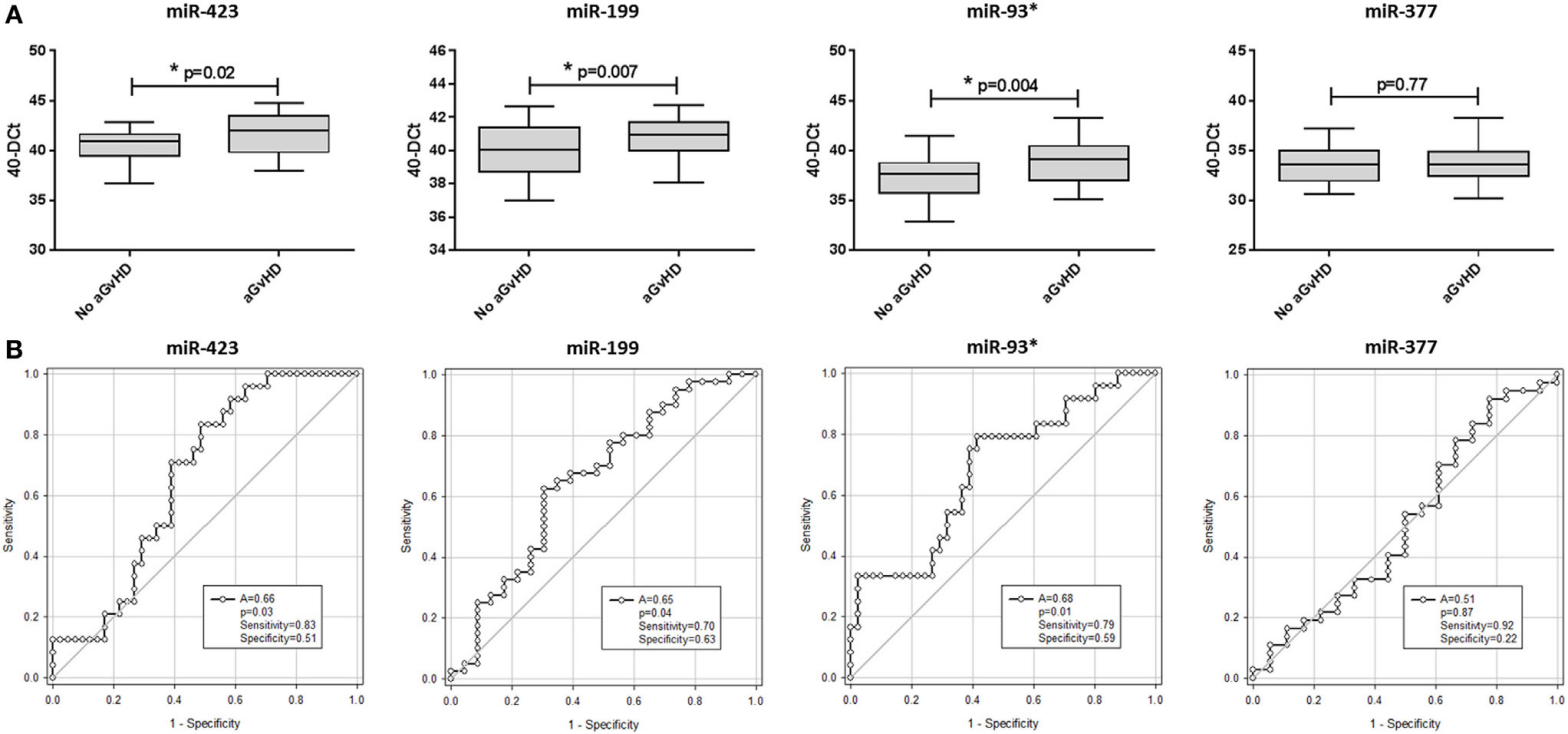
MicroRNA (miRNA) expression in diagnostic cohort patient serum samples. Expression of miR-423, miR-199, miR-93*, and miR-377 was assessed by quantitative real-time PCR at the onset of acute graft-versus-host disease (aGvHD) symptoms, or equivalent time points for patients who did not develop aGcvHD, in the diagnostic cohort (Vienna, *n* = 65) and analyzed according to aGvHD incidence. **(A)** miRNA expression at aGvHD diagnosis according to aGvHD incidence. Box plot whiskers represent minimum to maximum expression and *p*-values were calculated using the independent two sample *t*-test. **(B)** Receiver operator characteristic curves for incidence of aGvHD to determine diagnostic ability of miRNA expression. *p < 0.05.

In this diagnostic cohort, miR-423 (*p* = 0.02), miR-199 (*p* = 0.007), and miR-93* (*p* = 0.004) were expressed at a significantly higher level at aGvHD onset (Figure 2A). There was no differential expression of miR-377 at the onset of aGvHD (*p* = 0.77) (Figure 2A). None of the miRNAs could differentiate disease severity at onset, as there was no significant difference in expression between patients with mild (grades I–II) versus severe (grades III–IV) aGvHD (*p* > 0.05) (data not shown). According to ROC analysis, miR-423 (*p* = 0.03, AUC = 0.66), miR-199 (*p* = 0.04, AUC = 0.65), and miR-93* (*p* = 0.01, AUC = 0.68) but not miR-377 (*p* = 0.87, AUC = 0.51) had diagnostic ability with regard to aGvHD incidence (Figure 2B).

All three diagnostic miRNAs demonstrated significant positive correlation (miR-423 versus miR-199 *p* < 0.001, *R*^2^ = 0.38; miR-423 versus miR-93* *p* < 0.001, *R*^2^ = 0.54; and miR-199 versus miR-93* *p* < 0.001, *R*^2^ = 0.45) (data not shown). Thus, PCA was used to calculate a composite score and PC1 explained 78.2% of the variability. Composite ROC analysis based on PC1 [PC1 = (0.57853 * *Std*miR423) + (0.55938 * *Std*miR199) + (0.59363 * *Std*miR93*)] showed the three miRNA model to have diagnostic ability with respect to aGvHD incidence (*p* = 0.019, AUC = 0.68) (Figure S3B in Supplementary Material).

### Candidate miRNA Expression in Sequential Serum Samples

MicroRNAs were assessed in serum samples taken at sequential time points (pre-HSCT, D0, D7, D14, and D28) from a subset of patients of the prognostic cohort to analyze circulatory expression patterns during early HSCT. This subset comprised *n* = 34 HSCT patients [aGvHD *n* = 19 (56%): grades I = 5 (15%), II = 8 (24%), and III = 6 (17%); no aGvHD *n* = 15 (44%)] (Table S1 in Supplementary Material).

**Figure 3:**
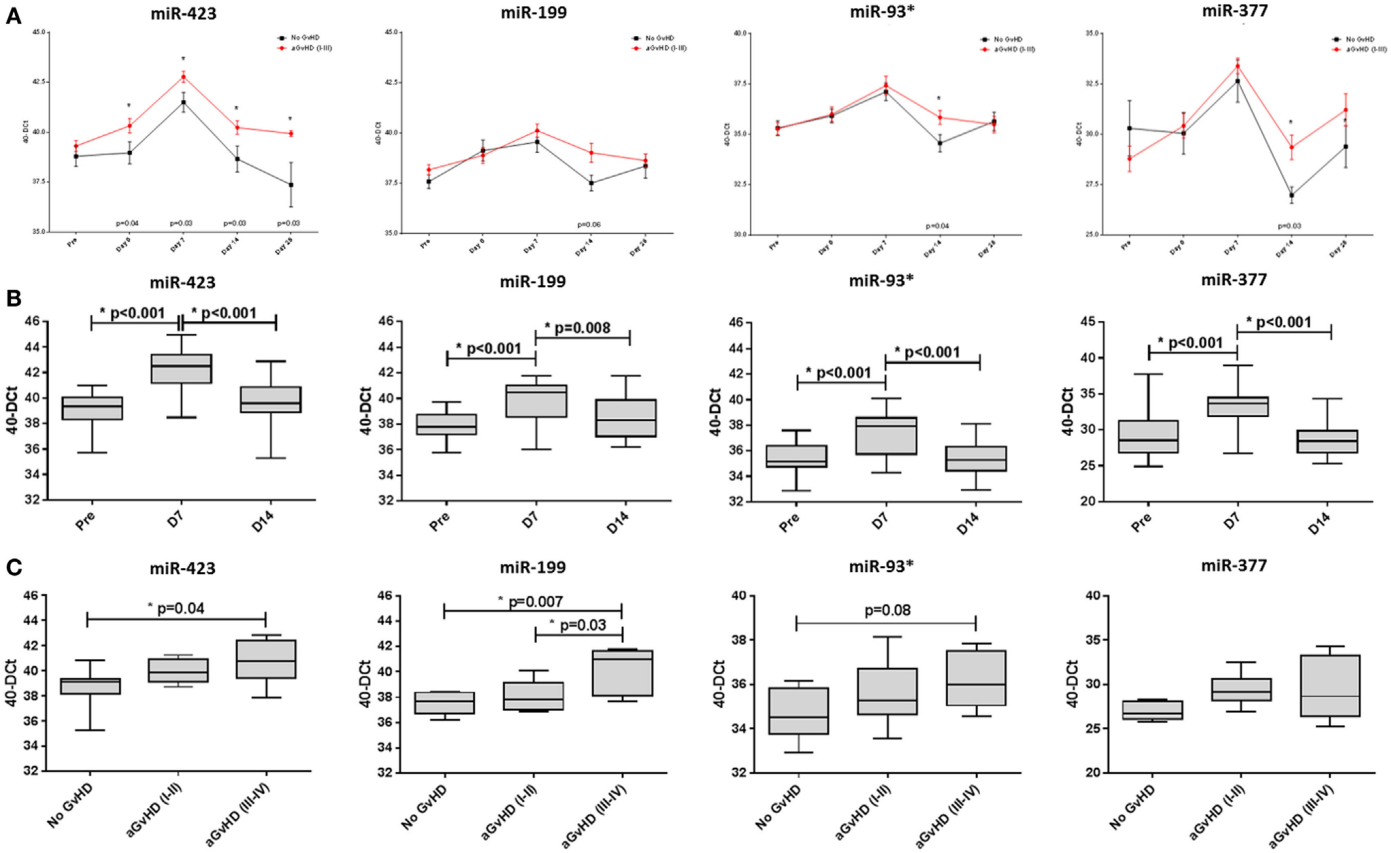
MicroRNA (miRNA) expression in sequential cohort patient serum samples. Expression of miR-423, miR-199, miR-93*, and miR-377 was assessed by quantitative real-time PCR and analyzed according to acute graft-versus-host disease (aGvHD) **(A,B)** incidence and **(C)** severity at sequential time points in *n* = 34 hematopoietic stem cell transplantation (HSCT) patients. **(A)** Patients were segregated based on the incidence of aGvHD and miRNA expression plotted at each time point. Differential miRNA expression between patients who developed aGvHD versus those who remained disease-free was assessed and significant or borderline differences are shown. Error bars represent mean with SEM and *p*-values were calculated using the *t*-test. **(B)** miRNA expression according to time point pre- and post-HSCT. Box plot whiskers represent minimum to maximum expression and *p*-values were calculated using the independent two sample *t*-test. **(C)** miRNA expression at day 14 according to aGvHD severity. Box plot whiskers represent minimum to maximum expression and *p*-values were calculated between groups using one-way ANOVA with Tukey *post hoc* multiple comparisons adjustment. *p < 0.05.

Assessing miRNA expression according to HSCT time points, miR-423 levels were elevated from the day of transplant (D0) to D28 (D0 *p* = 0.04, D7 *p* = 0.03, D14 *p* = 0.03, and D28 *p* = 0.03) in aGvHD patients compared with those who remained aGvHD-free, while miR-199, miR-93*, and miR-377 expression levels were elevated by D14 (*p* = 0.06, *p* = 0.04 and *p* = 0.03, respectively) in patients who later developed aGvHD (Figure 3A).

In all patients, miRNA expression in serum increased from pre-HSCT to D7 post-HSCT (miR-423 *p* < 0.001, miR-199 *p* < 0.001, miR-93* *p* < 0.001, and miR-377 *p* < 0.001), followed by a decrease in expression at D14 (miR-423 *p* < 0.001, miR-199 *p* = 0.008, miR-93* *p* < 0.001, and miR-377 *p* < 0.001) (Figure 3B).

In relation to aGvHD severity, miR-423, miR-199, and miR-93* were differentially expressed at D14 according to subsequent aGvHD grade. Specifically, miR-423 was expressed at a significantly higher level in patients later developing aGvHD (grades III–IV) versus no GvHD (*p* = 0.04) and miR-199 expression was significantly higher in patients subsequently experiencing aGvHD (grades III–IV) versus no GvHD (*p* = 0.007) and versus aGvHD (grades I–II) (*p* = 0.03) (Figure 3C). Similarly, miR-93* expression was higher in patients with subsequent aGvHD (grades III–IV) versus no aGvHD (*p* = 0.08) (Figure 3C). MIR-377 expression appeared to increase according to aGvHD grade, but the differences were not statistically significant (Figure 3C).

### Candidate miRNA Expression in Sequential EV Samples

**Figure 4:**
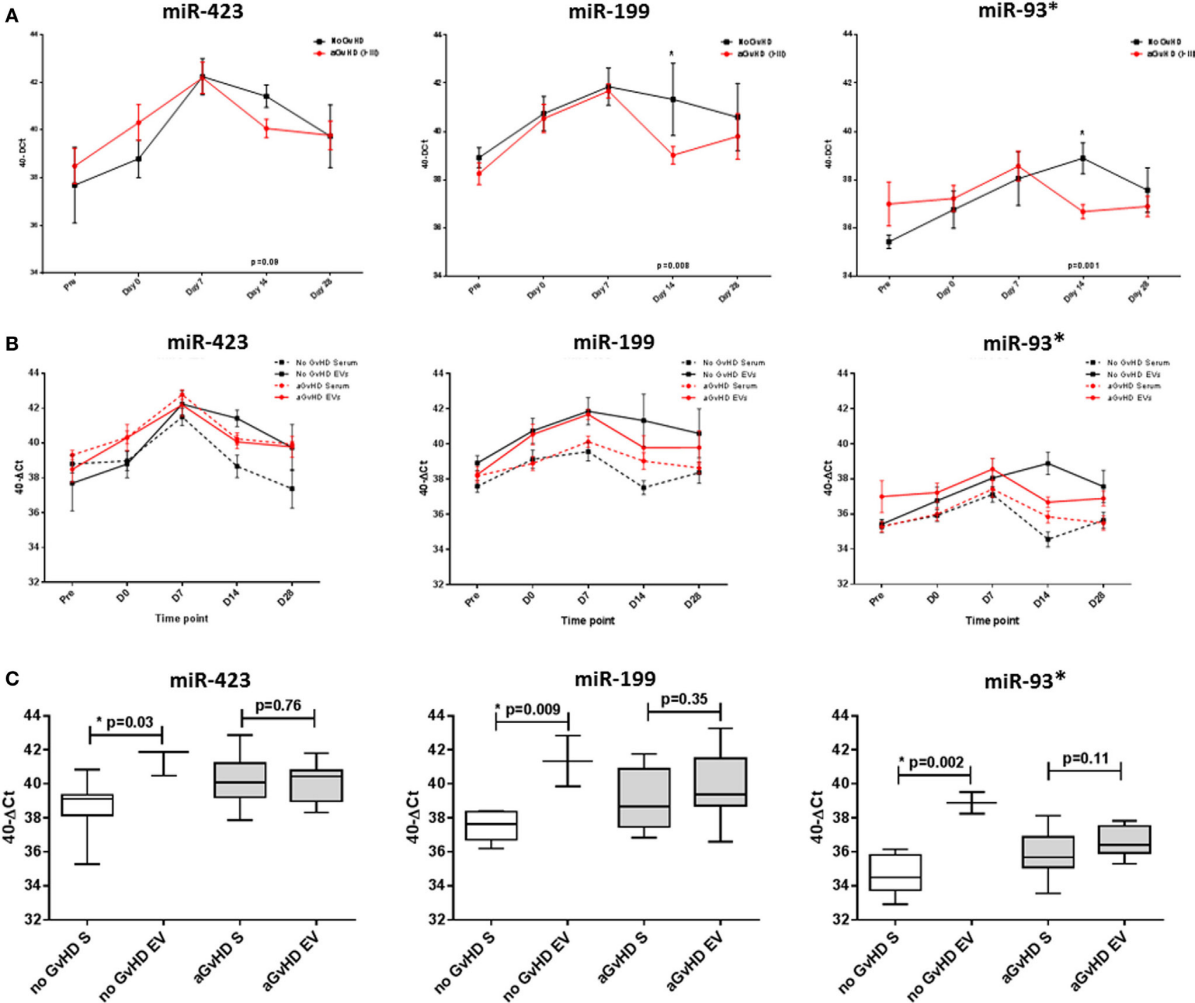
MicroRNA (miRNA) expression in sequential cohort patient extracellular vesicle (EV) and matched serum samples. Expression of miR-423, miR-199, and miR-93* was assessed by quantitative real-time PCR within serum and EVs and analyzed according to acute graft-versus-host disease (aGvHD) incidence in a sequential time points cohort (*n* = 15). **(A)** Patients were segregated based on the incidence of aGvHD, and EV miRNA expression plotted at each time point. Differential miRNA expression between patients who developed aGvHD versus those who remained disease-free was assessed and significant or borderline differences are shown. Error bars represent mean with SEM and *p*-values were calculated using the independent two sample *t*-test. **(B)** Comparisons between miRNA expression in EVs and matched serum samples (*n* = 15) at sequential time points, segregating patients based on the incidence of aGvHD. **(C)** miRNA expression at day 14 according to aGvHD incidence in whole serum (S) and the EV fraction (*n* = 15). Box plot whiskers represent minimum to maximum expression and *p*-values were calculated using paired *t*-tests. *p < 0.05.

In order to investigate the candidate miRNAs within the EVs of serum, expression was assessed at sequential time points during early HSCT (pre-HSCT, D0, D7, D14, and D28), in a subset of 15 patients (Table 1). miRNA EV expression was lower at D14 in patients who later developed aGvHD compared with those who did not and this was significant for miR-199 (*p* = 0.008), miR-93* (*p* = 0.001), and approaching significance for miR-423 (*p* = 0.09) (Figure 4A). There was no significant difference in expression of miR-377 according to subsequent aGvHD incidence (data not shown).

Expression of miR-423, miR-199, and miR-93* was compared between serum and the circulating EV fraction for matched samples (*n* = 15) taken at time points pre-HSCT to D28 post-HSCT (Figure 4B). Expression patterns showed similarities for earlier time points, however, by D14 patients who did not subsequently develop aGvHD had markedly higher expression of miR-423 (*p* = 0.03), miR-199 (*p* = 0.009), and miR-93* (*p* = 0.002) in the EV fraction compared with circulating serum, while expression was similar in patients who later developed aGvHD (Figure 4C).

### Candidate miRNA Expression in Verification EV Samples

Extracellular vesicle expression of miRNA data was replicated in an independent D14 verification cohort comprising *n* = 47 HSCT patients with higher grade aGvHD versus no aGvHD [aGvHD *n* = 24, grades I = 0 (0%), II = 22 (47%), and III = 2 (4%), no aGvHD *n* = 23 (49%)] (Table 1). There was no significant difference in patient age (*p* = 0.93), gender (*p* = 0.56), conditioning regimen (*p* = 0.11), relationship (*p* = 0.08), survival (*p* = 0.10), or follow up (*p* = 0.32) between patients who developed aGvHD (mean onset 38 days post-HSCT) compared with those that did not (Table 1).

**Figure 5:**
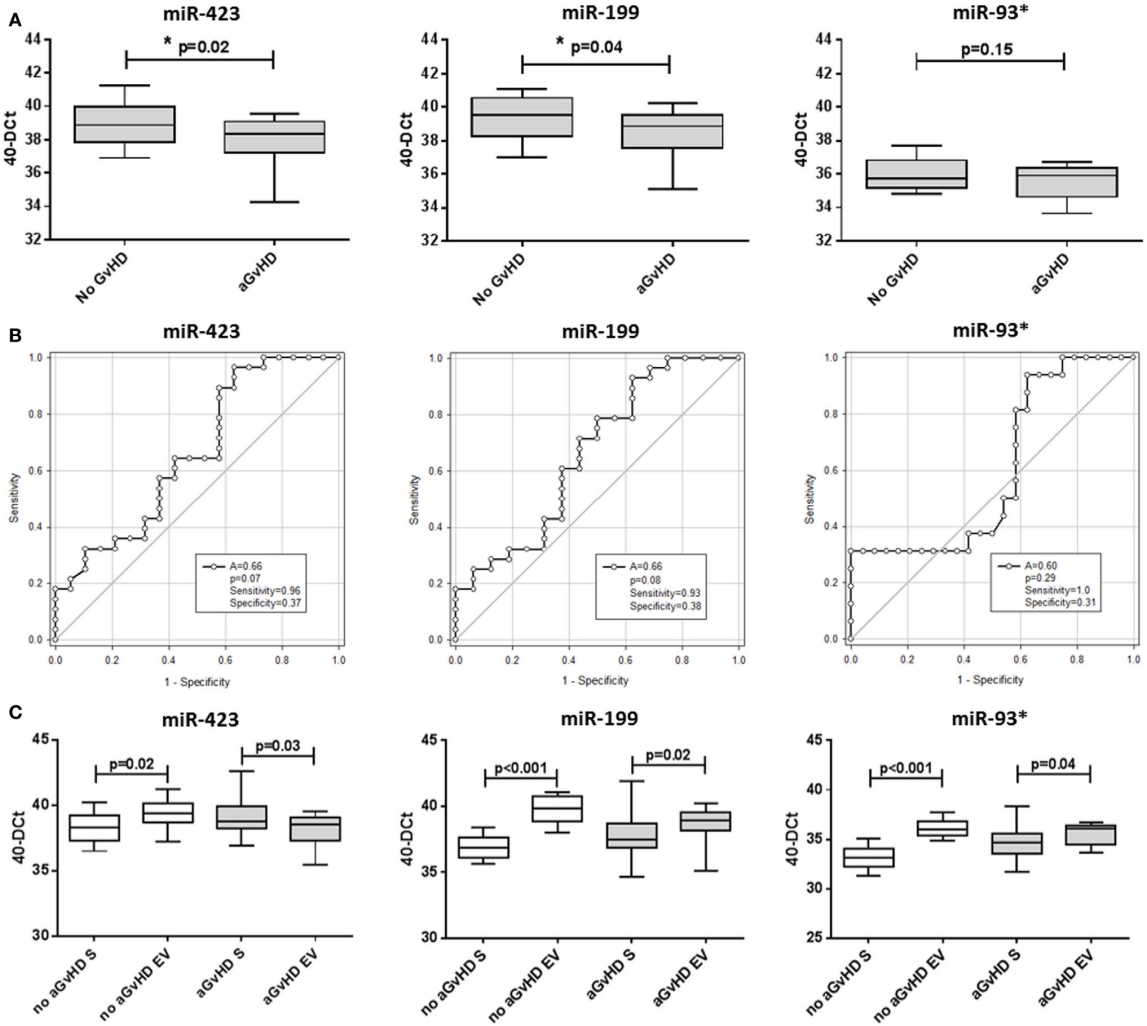
MicroRNA (miRNA) expression in verification cohort patient extracellular vesicle (EV) and matched serum samples. Expression of miR-423, miR-199, and miR-93* was assessed by quantitative real-time PCR within serum EVs and analyzed according to acute graft-versus-host disease (aGvHD) incidence in the day 14 (D14) verification cohort (n = 47). **(A)** miRNA expression in the EV verification cohort at D14 according to aGvHD incidence. Box plot whiskers represent minimum to maximum expression and *p*-values were calculated using the independent two sample *t*-test. **(B)** Receiver operator characteristic curves in the EV verification cohort for incidence of aGvHD to determine prognostic ability of miRNA expression. **(C)** miRNA expression at D14 according to aGvHD incidence in matched whole serum (S) and the EV fraction (*n* = 47). Box plot whiskers represent minimum to maximum expression and *p*-values were calculated using paired *t*-tests. *p < 0.05.

MicroRNA EV expression at D14 was decreased in patients who later developed aGvHD compared with aGvHD-free patients, and this was significant for miR-423 (*p* = 0.02) and miR-199 (*p* = 0.04), but not for miR-93* (*p* = 0.15) (Figure 5A). In ROC analysis, miR-423 (*p* = 0.07, AUC = 0.66) and miR-199 (*p* = 0.08, AUC = 0.66) were approaching significance for association with risk of aGvHD, while miR-93* did not demonstrate significant prognostic ability (*p* = 0.29, AUC = 0.60) (Figure 5B). Expression of miR-423 and miR-199 in D14 EVs was significantly positively correlated (miR-423 versus miR-199 *p* < 0.001, *R*^2^ = 0.55), while miR-423 and miR-93* demonstrated weak negative correlation (miR-423 versus miR-93* *p* = 0.02, *R*^2^ = 0.13) and miR-199 expression did not correlate with miR-93* expression (miR-199 versus miR-93* *p* = 0.19, *R*^2^ = 0.19) (data not shown). PC analysis was calculated using predictive miRNAs (miR-423/miR-199) and PC1 explained 68.8% of the variability in the data. Using PC1 [PC1 = (0.64068 * *Std*miR423) + (0.59539 * *Std*miR199)] for composite ROC analysis showed that miR-423 and miR-199 had prognostic potential (*p* = 0.06, AUC = 0.69) (Figure S3C in Supplementary Material).

miR-423, miR-199, and miR-93* expression data were compared between *n* = 47 matched D14 samples of the EV verification and serum verification cohort. Similar to the previous smaller subset analysis of 15 sequential patients, at D14 patients who subsequently did not develop aGvHD had significantly higher expression of miR-423 (*p* = 0.02), miR-199 (*p* < 0.001), and miR-93* (*p* < 0.001) in the EV fraction compared with circulating serum, while this difference was less marked in patients who later developed the disease (miR-423 *p* = 0.03, miR-199 *p* = 0.02, and miR-93* *p* = 0.04) (Figure 5C).

## Discussion

Recently, circulating miRNAs within the serum or plasma have been shown to act as robust biomarkers for a variety of diseases. In this study, expression of miR-423, miR-199, miR-93*, and miR-377 was assessed in circulating serum prior to aGvHD, at disease onset and at sequential HSCT time points, and associated with predicting disease incidence and severity. The study is initially replicatory in nature, in order to validate the results of previous reports demonstrating the potential of circulatory miRNAs as aGvHD biomarkers (7), and consequently extends these data into the novel field of EVs.

Unfortunately, GvHD has remained a frequent complication of HSCT. Diagnosis is typically based on clinical parameters confirmed by biopsy and thus, biomarkers are needed to aid in predicting disease onset and for objective diagnosis. Plasma biomarkers, particularly proteins, have shown promise (20). However, extensive validation is required to improve risk stratification or begin customized treatment, and protein-based methodology can be insensitive and unsuitable for clinical translation. In contrast, miRNAs are low in complexity, expressed specifically in different tissues, and biological stages, robust and can be accurately detected by common laboratory techniques (8, 21).

Xiao et al. reported that high expression of a four miRNA model (miR-377, miR-423, miR-199, and miR-93*) was associated with aGvHD onset and poor overall survival (7). As with all potential biomarkers, however, replication is required in independent cohorts to confirm reproducibility, especially when clinical protocols can be varied. We initially assessed the candidate miRNAs in an prognostic cohort of D14 post-HSCT serum samples and confirmed the results of Xiao et al., demonstrating significantly elevated expression of miR-423, miR-199, miR-93*, and miR-377 in patients who subsequently developed aGvHD, and their prognostic ability by ROC analysis. Xiao et al. also assessed miRNA expression in matched patient samples taken before the onset of aGvHD (2 weeks post-HSCT) and after the onset of aGvHD symptoms (6 weeks post-HSCT) and observed higher expression after aGvHD had occurred (7). This may appear to be contradictory with results of the sequential time point expression analysis performed in our study, where we assessed miRNA expression at 7-day intervals until 28 days post-HSCT and observed highest expression levels at day 7, which then decreased in all patients regardless of aGvHD status. However, it is difficult to make a direct comparison between the data sets without having a direct comparison of time points. Indeed, expression at the earlier 7-day point was not compared in the Xiao et al. study, and additionally, the last time point assessed in our sequential analysis was D28 post-HSCT, while patients developed aGvHD at a mean onset of 42 days (prognostic cohort) and 34 days (diagnostic cohort) post-HSCT. According to our data, although miRNA expression decreased in all patients between 7 and 14 days post-HSCT, levels between 14 and 28 days post-HSCT remained similar, and increased in the case of miR-377. Is it not inconceivable that if a comparison was made between miRNA expression levels pre- and post-aGvHD time points in the present study, differences may be observed. Xiao et al. also reported an association between the combined miRNA model and poor OS (7). Although a significant association with OS was not identified in the present study, the model was correlated with NRM, suggesting the miRNAs may predict mortality from causes other than relapse, such as incidence of aGvHD. In an independent diagnostic cohort comprising serum samples taken at aGvHD onset, miR-423, miR-199, and miR-93* were also elevated, further demonstrating their dysregulated expression in aGvHD and important biomarker potential. Although data demonstrated by Xiao et al. was generated using plasma samples, studies comparing the plasma, and serum fractions of blood have suggested a high degree of correlation in miRNA expression (8, 9, 22). Thus, verifying the results of Xiao et al. in an alternative sample source further highlights the reproducibility of the data.

Xiao et al. hypothesized that the candidate miRNAs may play a role in the process of donor T-cells alloreactivity against recipient tissues, or in response to damage in target cells during aGvHD, *via* selective inclusion into multivesicular bodies or exosomes which are released into the blood stream. In the present study, the EV fraction of serum was investigated in sequential early HSCT time points and results replicated in a larger cohort of D14 post-HSCT samples. Although disadvantages have been reported with the choice of serum compared to plasma as an EV source, such as clot formation during the serum processing procedure affecting EV release, either by trapping EVs within the clot (23) or stimulating releasing platelet-derived EVs (24), serum samples are frequently utilized for optimization of EV isolation and analysis techniques (25–27). It has been suggested that plasma can be also be a challenging material, due to high levels of clotting factors interfering with EV precipitation reagents (PRs), leading to contaminating proteins, and microvesicles. While some reports suggest no difference in EV isolation from serum or plasma (23), additional studies are required in order to fully understand how EV expression might be affected by sample processing protocols (28). In this regard, the present study utilized a commercial PR for serum EV isolation. These reagents are relatively new to the market, and their specificity for EVs remains to be thoroughly validated. In a previous study, we demonstrated the LT PR to recover smaller vesicles than a competitor product, indicating improved selectively for small EVs (12). Other studies have suggested that EV pellets isolated using PR are free from contaminating cell organelles, including apoptotic bodies (29) and that the method recovers more EVs than ultracentrifugation (UC), chromatography, or magnetic beads, with higher EV RNA and protein purity (30). Interestingly, a report specific to serum samples demonstrated low levels of protein-bound miRNA contamination in EVs isolated using PRs (27). Although UC has historically been regarded as the gold standard for EV isolation, the practical application of this method is limiting with regard to the sample volumes required, length of isolation, and requirement for specialist equipment. To this end, PRs have been evaluated as a practically applicable alternative to UC in a clinical setting (31). However, despite the acceptance of PRs for EV isolation, caution must be applied and it would be incorrect to claim that the isolated EV pellets are entirely pure in nature.

The EV aspect of this study is novel and extends upon the hypotheses proposed by Xiao et al. EVs are known to regulate cellular differentiation of blood cells, metabolic pathways, and modulate immune function (3) and play a role in antigen presentation either directly, or indirectly *via* transfer to antigenic peptides or by cross-dressing APCs (32). Upon activation, T lymphocytes produce large amounts of EVs that bear TCR from the pool of activated complexes (33) and it is possible that sub-sets of T-cells may be further defined by their EV miRNome. Indeed, 20 intracellular miRNAs were shown to distinguish between sub-populations of CD4^+^ T-cells, defining the development, and differentiation of this lineage (34). Furthermore, CD4^+^CD25^+^Foxp3^+^ Tregs released EVs following activation in a mouse model (35) and CD8^+^CD25^+^Foxp3^+^ T-cells secreted EVs that inhibited DC-induced CD8^+^ CTL responses (36), demonstrating immune suppressive capacities for these EVs.

In the present study, expression of the candidate miRNAs (miR-423, miR-199, and miR-93*) was lower in the EV fraction of patients who subsequently developed aGvHD. In comparison with serum, miR-93* in the EV fraction did not show a strong association with aGvHD, suggesting this miRNA may not be differentially packaged into serum EVs in an HSCT setting. Patients who did not develop aGvHD had significantly higher levels of miRNAs within the EV fraction, and we hypothesize that these miRNAs are being specifically transported in the circulation to distal target organs, where they function in an aGvHD protective capacity. However, the relationship between miRNA expression in whole serum and the EV compartment is not completely understood, and the main source of circulatory miRNA carrier in serum is still debatable. While some groups have reported that the majority of miRNAs are included within EVs (37), there is also evidence to suggest that miRNAs are more commonly co-fractionated with protein complexes, such as Argo-2 (38, 39). To add further complication, it has also been shown that the mode of transport can be specific to individual miRNAs, for example an HDL, EV, or Ago2 carrier (39, 40), and the factors determining which mechanisms are used are also unclear. Finally, although it is well recognized that specific miRNA populations are selectively sorted in to EVs (41), current research suggests four potential mechanisms for this specific packaging [nSMase2-dependent pathway, hnRNPs-dependent pathway, the 3′end of the miRNA sequence-dependent pathway, and the miRISC-related pathway (42)], and understanding of these processes in a cell-, tissue-, bio-fluid-, and disease-specific context is unexplored. Altogether, while we have hypothesized explanations for the disparate miRNA expression between serum and EVs, this remains an area of ambiguity, but likely reflects the underlying function of individual miRNAs.

However, identifying the biological function of miRNAs can be challenging, due to their short seed sequence potentially targeting hundreds of mRNA transcripts. miR-423, miR-199, and miR-93* have suggested roles in inflammation, tissue damage, regulation of cell proliferation, and tissue repair, but their specific functions in an aGvHD setting are yet to be elucidated (7). miR-423 has been implicated as a circulating marker for heart failure (43) and promotes cell growth by regulating G_1_/S transition in hepatocellular carcinoma *via* p21Cip1/Waf1 (44). It has been postulated that cell cycle progression from the G_1_/S phase may be a critical step for activation of immune-related cells in the pathogenesis of GvHD. Thus, miR-423 expression observed in this study may function to regulate Waf1, affecting cell cycle progression. miR-199 has also been shown to regulate cell proliferation and survival, by targeting caveolin-2, a plasma membrane scaffolding protein involved in cellular growth control, and apoptosis (45). Interestingly, caveolin-1 has been shown to regulate TCR signal strength and Treg differentiation, whereby cav-1^−^/^−^ donor T-cells cause less severe GvHD and yield higher numbers of Tregs (46). Caveolin-1 and -2 share structural similarities and are commonly co-expressed (45), however, miR-199 regulation of caveolin-1 is less well established in non-cell line material. miR-199 has also been shown to regulate mTOR in malignant cells (47–50). In GvHD, suppression of mTOR by rapamycin inhibits the generation of Th17 cells and enhances TGF-β-induced generation of FoxP3^+^ Tregs (51). A low Th17/Treg ratio correlates with severe clinical and pathological GvHD, apoptosis intensity, and TNF-α expression (52). Accordingly, high levels of Th17 cells are associated with lethal GvHD in mice, hallmarked by extensive pathologic cutaneous, and pulmonary lesions (53), while a high percentage of Treg cells is known to ameliorate aGvHD (54). Taken together, this may suggest a protective role for miR-199, whereby increased EV expression leads to target cell mTOR suppression and a downstream reduction in aGvHD severity. miR-93* has been demonstrated to promote tumor growth and angiogenesis *via* targeting of integrin-B8 (encoded by *ITGB8*) (55). Mouse CD4^+^Foxp3^+^ Tregs express integrin-B8, which acts as a marker for thymic-derived Tregs. Functionally, Tregs from *ITGB8* knockouts (KO) exhibit normal suppressor function in *in vitro* and *in vivo* models of colitis, but fail to provide TGF-β1. *ITGB8* KO Tregs express higher surface TGF-β1 levels, consistent with defective processing (56), and *ITGB8* KO mice demonstrate no suppressive ability against the innate or adaptive immune response and fail to reduce the level of inflammatory cytokines (57). In breast cancer cell lines, Liu et al. demonstrated that miR-93* induces mesenchymal epithelial transition, associates with downregulation of multiple stem cell regulatory genes including JAK1, STAT3, and EZH1 (58). Activated JAK1 is required for T-effector cell responses in inflammatory disease, and inhibition leads to improved survival of mice following aGvHD, reduced histopathological grading, and lower serum pro-inflammatory cytokine levels (59). Associations with STAT3 may also have implications in aGvHD pathology, whereby the STAT3 transcription factor promotes Treg instability and is increased prior to aGvHD onset (60, 61). EZH1 is also implicated in mediating GvHD, whereby conditional loss of EZH2 in donor T-cells inhibits GvHD in mice, specifically impairing T-cell differentiation into IFN-gamma producing effector cells (62). Studies in kidney have shown that miR-93* also targets vascular epithelial growth factor-A (VEGF) (63), a cytokine associated with hematopoietic cancer outcome, immune modulation, and tissue repair (64, 65). With regard to GvHD, VEGFA levels have been associated with incidence of aGvHD (66), relapse, and non-relapse mortality following HSCT (64). miR-377 has been shown to suppresses cell proliferation by targeting CDK6, a cyclin-dependent kinase that regulates G1/S progression, and is highly overexpressed in patients with GvHD (67). Interestingly, it was demonstrated that miR-377 expression is regulated by CyA, an immunosuppressant drug commonly administered for GvHD prophylaxis, and that miR-377 inhibition resulted in reduced CyA-mediated apoptosis (68). It would be interesting to assess the effect of CyA on miR-377 expression in serum. In the present study, miR-377 was not replicated as a reproducible predictive biomarker and interestingly, all Newcastle patients with available information on aGvHD prophylaxis received CyA.

The increased miRNA expression observed at day 7 post-HSCT was an interesting finding of this study, particularly as levels remained higher in the patients who subsequently developed aGvHD. We did not detect any significant differences in the conditioning regimens between patients who developed aGvHD compared with those who did not, and so this cannot explain the data. We hypothesize that elevated miRNA expression at day 7 in both aGvHD and no aGvHD patients is a reflection of an elevated inflammatory reaction, in response to the initiation of HSCT conditioning regimens, and the HSCT procedure. It is well established that mucosal barrier injury, induced by intensive cytotoxic therapy prior to HSCT, initiates the release of pro-inflammatory cytokines, chemokines, antimicrobial peptides, and the acute phase protein, LPS-binding protein (LBP) (69, 70). Interestingly, serum expression levels of inflammatory markers (CRP, IL-8, and LBP) peak 20 days following cytotoxic conditioning therapy, which corresponds to 1-week post-HSCT (70, 71). Furthermore, a range of miRNAs are significantly elevated in serum post-chemotherapy (72). miRNAs have been established as key regulatory factors in wide ranging elements of immune function including homeostasis, response, tolerance, and auto-immunity. Furthermore, their functionality may be achieved by multiple arrangements, including a linear model (one miRNA controlling one target), divergent model (single miRNA controlling multiple targets with distinct biological outcomes), network model (coordinated regulation of multiple targets with one outcome), and convergent model (many targets coordinated by cognate miRNAs in a linear manner, contributing additively to a specific response) (73). Taken together, this highlights the significance of miRNAs as regulatory elements in the context of immune response, and the complexity surrounding trying to decipher their expression and specific function in relation to immune response activity.

As discussed, the miRNAs examined in the present study have previously been associated with inflammation, tissue damage, regulation of cell proliferation, and tissue repair and thus, it is not inconceivable that they may be among the spectrum of miRNAs elevated post-conditioning. Although it is not clear why these miRNAs remain elevated in the sub-group of patients who develop aGvHD compared with those who remain aGvHD-free, it is possible that this is a reflection of the escalating inflammatory cascade that occurs in patients who develop aGvHD. Indeed, elevated circulatory miRNA expression may initially be driven by the conditioning and transplant procedure-initiated inflammatory reaction, which subsequently manifests into a cytokine storm and tissue damage, induced by alloreactive T-cells of donor origin in patients who develop aGvHD, leading to sustained miRNA expression. Conversely, the inflammatory reaction diminishes in patients who do not develop aGvHD and miRNA expression may also be reduced, if not further stimulated by the allogeneic response mediated by donor T-cells. This would be an interesting area for further investigation. Although Sun et al. performed studies in mice to examine miRNA expression patterns in allogeneic T-cell responses, they did not detect significant expression of the miRNAs reported in the present study, despite homologs being present on the miRNA array (74). However, their study focused on allostimulated T-cells *in vitro*, while the present study concentrated on circulatory miRNA expression *in vivo*. Furthermore, differences in miRNA expression and function between mouse and clinical models cannot be ruled out. However, this does highlight the importance of comprehensive miRNA expression profiling in the aGvHD setting, which takes into account both the full array of recognized miRNAs and the heterogeneity in patient transplant protocols. Indeed, in a recent study we performed global expression profiling of *n* = 799 mature miRNAs in the serum of aGvHD patients (*n* = 12) at aGvHD diagnosis (75), which incorporated an expanded repertoire of miRNAs compared with the *n* = 345 species profiled by Xiao et al. (7). Although differential expression of the miR-423 and miR-199 (miR-93* was not present on the codeset) candidate miRNAs was confirmed, additional species with highly significant differential expression were also identified, suggesting that a panel of signature miRNA biomarkers may be a more potent predictor of aGvHD incidence and severity for this complex disease.

In conclusion, this study has replicated circulating miR-423, miR-199, and miR-93* as diagnostic and prognostic biomarkers for aGvHD in independent cohorts. Principal component analysis of miR-423, miR-199, and miR-93* confirmed the utility of the miRNA triad in predicting occurrence of aGvHD. In addition, for the first time we have demonstrated the expression of these miRNAs in the EV fraction, and results suggest patients who do not develop aGvHD have a higher level within serum EVs at D14, proposing a novel predictive biomarker role for EVs. The source of dysregulated miRNA expression remains unclear, particularly between donor-derived hematopoietic stem cells and patient-derived immune response cells, and this represents an important area for further investigation. Further assessment of the biological role of these miRNAs, particularly in the EV compartment, should inform on their contribution to the development of aGvHD.

## Ethics Statement

This study was carried out in accordance with the recommendations of the Newcastle and North Tyneside I Research Ethics Committee and the Ethics Committee of the Medical University of Vienna, Austria. All subjects gave written informed consent in accordance with the Declaration of Helsinki.

## Author Contributions

RC designed and performed the experiments, evaluated the data, and wrote the manuscript; JN assisted with experiments and drafting of the manuscript; MJ performed replication experiments in diagnostic samples and LB helped establish qRT-PCR protocols; KP advised and performed statistical analysis; CL performed clinical data collection and advised on PCA analysis; MC and HG contributed to clinical samples, data collection, and preparation of the manuscript; and AD developed the overall concept, supervised the research, and prepared the manuscript. All authors approved the final manuscript.

## Conflict of Interest Statement

The authors declare that the research was conducted in the absence of any commercial or financial relationships that could be construed as a potential conflict of interest.
