# Supplementary material for: Serum and Extracellular Vesicle MicroRNAs miR-423, miR-199, and miR-93* As Biomarkers for Acute Graft-versus-Host Disease
Source: Front Immunol. 2017 Nov 10;8:1446. doi: 10.3389/fimmu.2017.01446 (PMC5686047; doi:10.3389/fimmu.2017.01446)

Supplementary Figure 1. Serum EV isolation validation by Nanoparticle Tracking Analysis (NTA) and electron microscopy. (A) Size distribution curve of isolated serum EVs indicating the mean EV size is of the expected range. (B) Electron microscopy image of isolated Evs demonstrating the correct vesicle diameter range.

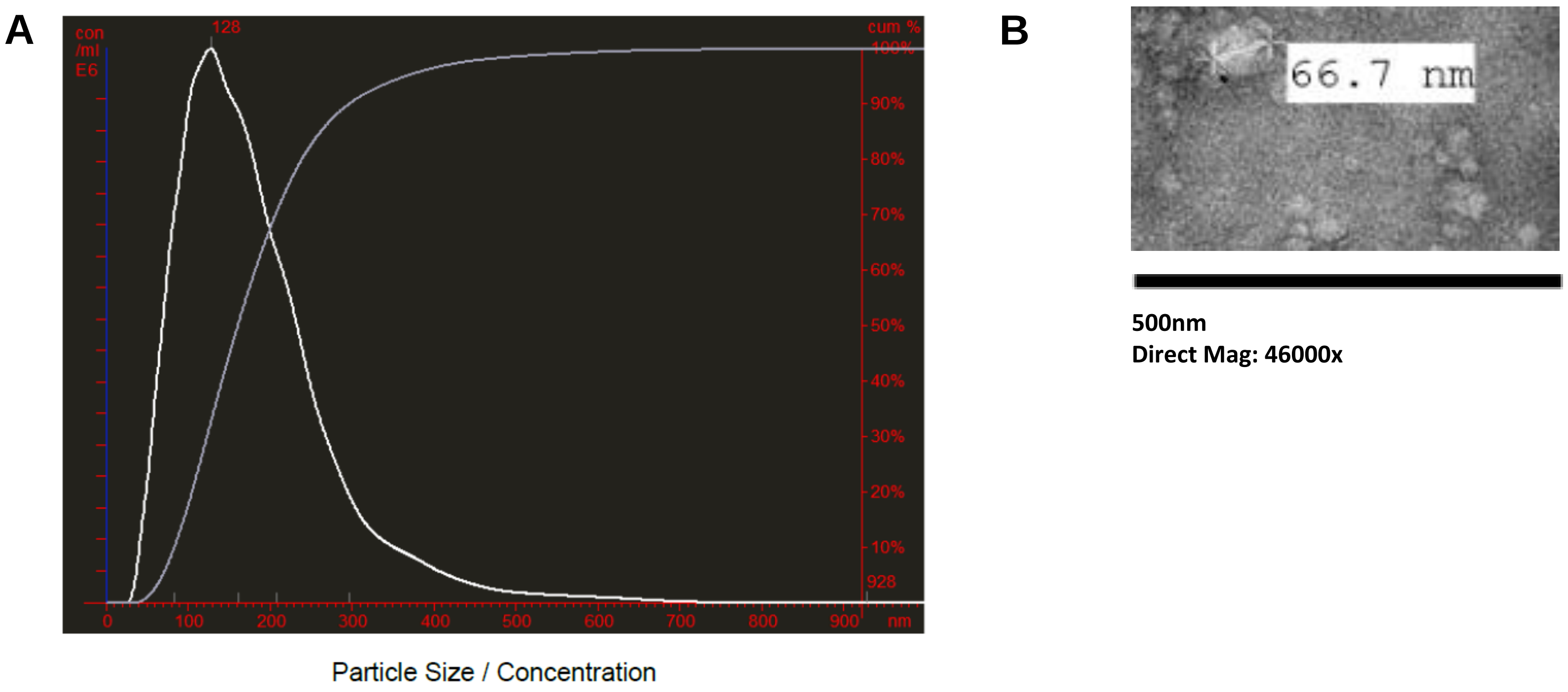

Supplementary Figure 2. MicroRNA correlation in prognostic serum cohort samples. Expression of miR-423, miR-199, miR-93* and miR-377 at D14 was assessed for expression correlation (n=81) (R^2;p-value). A linear regression line and p-values were calculated for Pearson's correlation coefficient. All p-values remained significant following Holm-Bonferroni multiple testing correction.

	**miR-199**	**miR-93***	**miR-377**	**miR-423**
**miR-199**		0.25; <0.001	0.08; 0.031	0.37; <0.001
**miR-93***	0.25; <0.001		0.16; 0.001	0.52; <0.001
**miR-377**	0.08; 0.031	0.16; 0.001		0.18; 0.001
**miR-423**	0.37; <0.001	0.52; <0.001	0.18; 0.001	

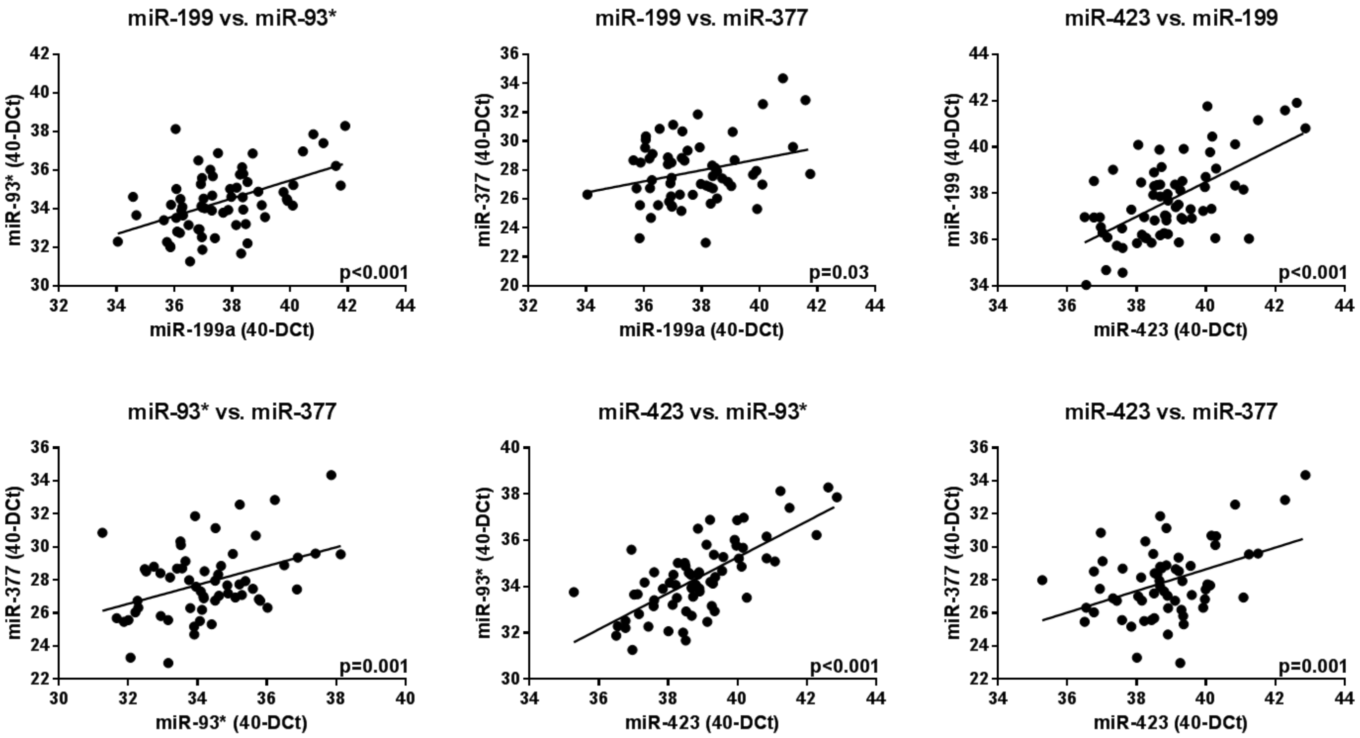

Supplementary Figure 3. ROC and NRM analysis using principle component 1 as a composite score. Principle component 1 was a linear combination of miR-93* and/or miR-423 and miR-199. (A) Receiver operator characteristic curve for principle component 1 (including miR-423, miR-199 and miR-93*) in the prognostic serum cohort (n=81) to determine prognostic ability for incidence of aGvHD. (B) Receiver operator characteristic curve for principle component 1 (including miR-423, miR-199 and miR-93*) in the diagnostic cohort (n=65) to determine diagnostic ability for incidence of aGvHD. (C) Receiver operator characteristic curve for principle component 1 (including miR-423 and miR-199) in the EV verification cohort (n=47) to determine prognostic ability for incidence of aGvHD. (D) Non-relapse mortality (NRM) according to principle component 1 (including miR-423, miR-199 and miR-93*) in the prognostic serum cohort (n=81), in relation to time from transplant to event. Dichotomised PC1 in relation to relapse and NRM is depicted by dotted and solid black and grey lines, respectively.

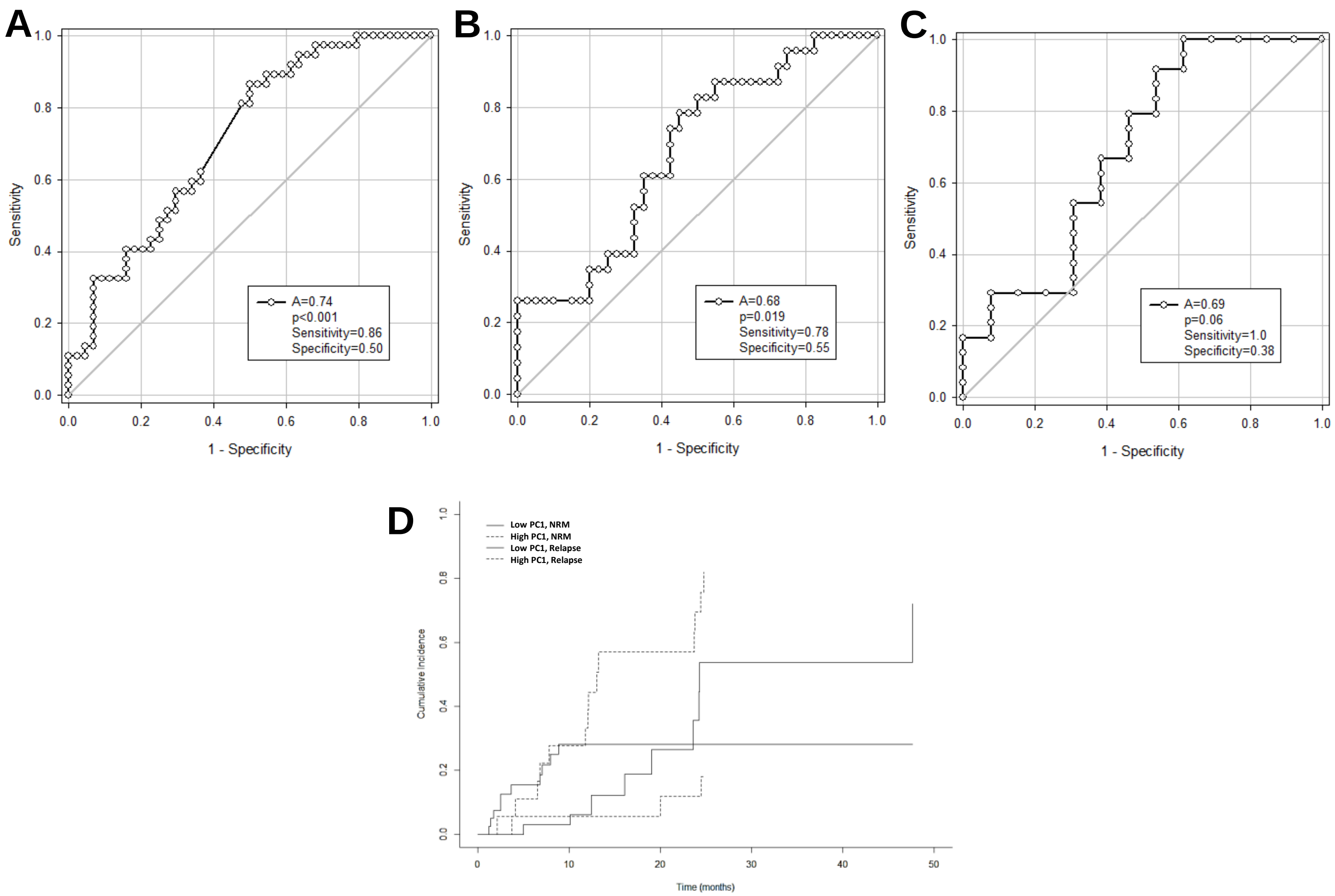

Supplement: Supplementary file 1 [file image_1.pdf]
