# Supplementary material for: Serum and Extracellular Vesicle MicroRNAs miR-423, miR-199, and miR-93* As Biomarkers for Acute Graft-versus-Host Disease
Source: Front Immunol. 2017 Nov 10;8:1446. doi: 10.3389/fimmu.2017.01446 (PMC5686047; doi:10.3389/fimmu.2017.01446)

**Supplementary Table 1. Patient clinical characteristics.** Clinical details of the sequential serum cohort (n=34) to test for differences between aGvHD and no aGvHD groups. p-values between groups were calculated using the independent 2 sample t-test **^1^** or Fishers Exact test **^2^**, as appropriate. *MUD=matched unrelated donor, SIB=sibling donor.*


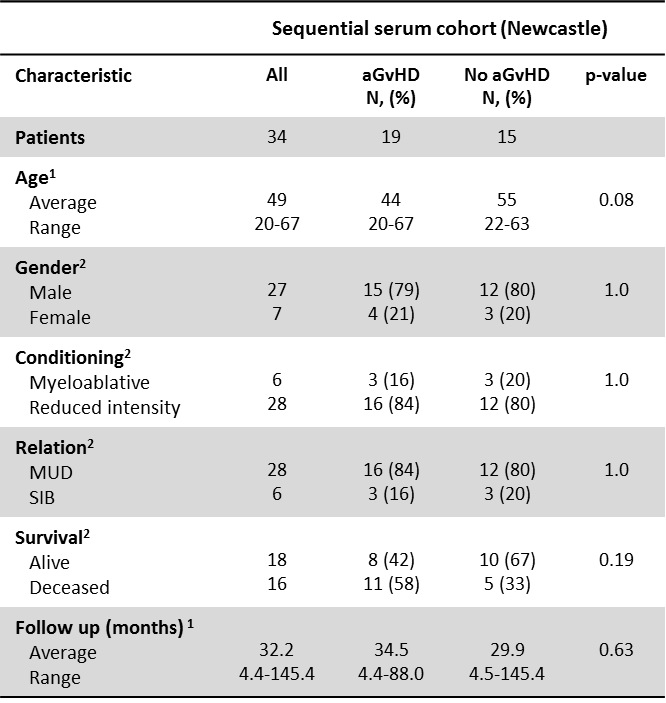

Supplement: Supplementary file 2 [file table_1.docx]
